# Recombinant *Bifidobacterium longum* Carrying Endostatin Protein Alleviates Dextran Sodium Sulfate-Induced Colitis and Colon Cancer in Rats

**DOI:** 10.3389/fmicb.2022.927277

**Published:** 2022-06-30

**Authors:** Zhiqian Bi, Enqing Cui, Yingying Yao, Xiaoyao Chang, Xiaoyang Wang, Yuhui Zhang, Gen-Xing Xu, Hongqin Zhuang, Zi-Chun Hua

**Affiliations:** ^1^The State Key Laboratory of Pharmaceutical Biotechnology, College of Life Sciences, Nanjing University, Nanjing, China; ^2^Changzhou High-Tech Research Institute of Nanjing University, Changzhou, China; ^3^Jiangsu Target Pharma Laboratories Inc., Changzhou, China; ^4^School of Biopharmacy, China Pharmaceutical University, Nanjing, China

**Keywords:** inflammatory bowel disease, *Bifidobacterium*, endostatin, colon cancer, gut microbiota

## Abstract

*Bifidobacterium* has been widely administrated orally as probiotics to prevent pathogen colonization and modulate the gut microbiome balance. Endostatin is an endogenous inhibitor of angiogenesis and has been shown to inhibit tumor growth, invasion, and metastasis. At present, the combination of endostatin and chemotherapeutic drugs has been regarded as a promising antitumor treatment strategy. In this study, we selected a safe strain of *Bifidobacterium longum* as a delivery system to transport endostatin to the gastrointestinal tract and explored their combined effect on inflammatory bowel disease (IBD) and colitis-associated cancer. The results indicated that *B. longum-Endo* relieved dextran sulfate sodium-induced body weight loss, diarrhea, colon shortening, and epithelium damage. Long-term oral administration of *B. longum-Endo* significantly decreased tumor formation rate, tumor number, and tumor size. Moreover, the effect of *B. longum-Endo* on gut microbiota dysbiosis was also confirmed by 16S rRNA sequencing analysis. The levels of potentially beneficial bacteria, such as *Lactobacillus*, *Bifidobacterium*, *Allobaculum*, and *Parabateroides*, were increased in the *B. longum-Endo* group compared to the model and *B. longum* groups. Meanwhile, levels of potentially pathogenic bacteria including *Desulfovibrio*, *Helicobacter*, and *Enterorhabdus* were decreased. Taken together, these results suggested that oral administration of recombinant *B. longum-Endo* strain may be a promising therapeutic strategy for IBD and colitis-associated cancer.

## Introduction

Inflammatory bowel disease (IBD), including Crohn’s disease (CD), and ulcerative colitis (UC), is characterized by chronic and recurrent mucosal inflammation of the gastrointestinal tract (Molodecky et al., 2012; Flynn and Eisenstein, 2019). Although the incidence of IBD is increasing, its cause remains poorly understood (Kaplan, 2015; Agrawal et al., 2022; Rana et al., 2022). Accumulating evidence indicated the importance of gut microbiota, whose disorder is a hallmark of IBD (Alkadhi et al., 2014). Clinically, the gut flora of IBD patients differs dramatically from healthy ones. For example, IBD patients exhibited diminished diversity of their gut microbiome, expansion of pro-inflammatory bacteria like *Enterobacteriaceae* and *Fusobacteriaceae*, and depletion of anti-inflammatory bacteria such as *Firmicutes* (Andoh et al., 2011; Ni et al., 2017; Burrello et al., 2018). Moreover, long-standing chronic inflammation and gut microbiota dysregulation promote IBD development into colon cancer. Clinical studies revealed that regaining the balance of intestinal microbial vial oral supplementation of probiotic bacteria is a promising way to prevent IBD and CRC (Sanders et al., 2013; Abreu and Peek, 2014).

*Bifidobacteria* are anaerobic Gram-positive bacteria that belong to the natural inhabitant of the intestine. Their presence in the human gastrointestinal tract is often associated with health benefits, such as treating and preventing gastrointestinal disorders (Kamada et al., 2013; Fouhy et al., 2019), reducing lactose intolerance (Guerreiro et al., 2014), enhancing immune system (Klaenhammer et al., 2012), preventing and reducing colonization of the gastrointestinal tract by *Helicobacter pylori* (Miki et al., 2007). *Bifidobacterium longum*, belonging to the *Bifidobacterium* genus, has become increasingly attractive in pharmaceutical and dairy products due to their safety and efficacy (Gaggia et al., 2011). It appears in the gut shortly after birth and can be detected throughout an individual’s lifespan. When the normal bacterial flora is disturbed, exogenously supplemented *B. longum* can rapidly colonize the intestinal tract and strengthen gut barrier function (Lewis et al., 2015; Schroeder et al., 2018). Many studies have shown that orally administration of *B. longum* could treat CD and UC (Miyauchi et al., 2013; Jang et al., 2018). Recently, it has been shown that *B. longum* could be used as a biotherapeutic agent for the treatment of colorectal cancer (CRC; Singh et al., 1997; Fahmy et al., 2019; Valadez-Bustos et al., 2019). Additionally, *B. longum* exhibited a potent tumor-targeting capability. Yazawa et al. (2000) used *B. longum* as a delivery system for gene therapy on tumor-bearing mice. Our previous study showed that using *B. longum* as the delivery system could selectively inhibit angiogenesis and hypoxic tumor growth (Hu et al., 2009). Currently, *B. longum* as a carrier to express protein or polypeptides with specific functions has become a new therapeutic way for cancer.

Angiogenesis is a tightly regulated process by a network of pro- and anti-angiogenic factors (Carmeliet and Jain, 2000; Italiano et al., 2008). Due to their critical role in promoting tumor growth and metastasis, anti-tumor angiogenesis has become an important cancer therapy target (Li and Cozzi, 2010; Hanahan and Weinberg, 2011). Endostatin is a 20 kDa carboxy-terminal fragment of collagen XVIII and has a strong effect on tumor growth inhibition (Perkins et al., 2009). Several researches showed that endostatin exhibited broad-spectrum antitumor activity in many cancers, such as liver cancer, breast cancer, gastric cancer, and CRC (Ma et al., 2004; Chen et al., 2013; Li et al., 2015). To date, recombinant human endostatin (Endostar) has been approved to treat non-small cell lung cancer. Clinically, the combination of endostatin and chemotherapeutic drugs has been regarded as a promising antitumor treatment strategy. In this study, we selected a strain of *B. longum* as the delivery system for endostatin protein to treat IBD and colitis-associated CRC.

## Materials and Methods

### Cloning and Expression of Endostatin in *Bifidobacterium longum*

Wild-type *B. longum* strain ATCC 55813 was preserved in our laboratory and anaerobically cultured at 37°C in MRS medium supplemented with 0.05% (w/v) L-cysteine. *Escherichia coli* DH5α was purchased from ATCC and cultured in Luria-Bertani medium. pBV222, a shuttle vector, was kindly provided by the Institute of Virology, China College of Preventive Medical Sciences. The Human endostatin gene was synthesized by Nanjing Genscript Biotech Corporation (China) from the human liver cDNA library. The construction of pBV222-Endo has been described previously (Li et al., 2003). The pBV222 and pBV222-Endo plasmids were transformed directly into *B. longum* strain ATCC 55813 by electroporation using a Bio-Rad Gene Pulser. Transformed *B. longum* was screened on TPY agar plates containing 0.5M sucrose and 5 mg/mL CM in anaerobic conditions. Recombinant human endostatin was separated by SDS-PAGE and detected by western blotting. Anti-Endostatin antibodies (Oncogene Research Products, San Diego, United States) were used for western blotting analysis, and the specific preparation was performed according to articles previously reported (Li et al., 2003; Xu et al., 2007).

### Experimental Animals and Ethics Approval

All C57BL/6J mice were purchased from Shanghai Laboratory of Animal Center (Shanghai, China) and housed in a temperature-controlled sterile room. Animal welfare and experimental procedures strictly followed high standard animal welfare and related ethical regulations approved by Nanjing University Animal Care and Use Committee.

### Induction and Treatment of Dextran Sulfate Sodium-Induced Colitis

As shown in Supplementary Figure 1, we designed the following acute colitis experimental protocol. 3.0% (w/v) dextran sulfate sodium (DSS; molecular weight, 36–50 kDa, MP Biomedicals) dissolved in drinking water was administered *ad libitum* for seven consecutive days. All mice were randomly divided into the following groups [*n* = 6–8 for each group]: (1) Control group, in which mice only received tap water without DSS; (2) Model group, in which mice received 3% DSS by drinking water for 7 days, and orally administered daily with 5% glucose in 0.9% NaCl; (3) *B. longum* group, in which mice were induced colitis by 3% DSS and orally administered daily with *WT B. longum*; and (4) *B. longum-Endo* group, in which mice were induced colitis by 3% DSS and orally administered daily with *B. longum-Endo.* Before gavage, *B. longum* and *B. longum-Endo* were washed three times and resuspended with 5% glucose in 0.9% NaCl. Each mouse was orally administrated with 200 μL (1.5 × 10^10^ CFU/kg/mouse) of the above suspension and carried out once daily for 7 days continuously. In the recovery experiment period, mice were fed with 3% DSS for 7 days and then changed to sterile water for additional 5 days. Oral administration with *B. longum* (1.5 × 10^10^ CFU/kg/mouse) or *B. longum-Endo* (1.5 × 10^10^ CFU/kg/mouse) was performed once daily for 12 days. The average water and DSS consumption per mouse per day were the same for all groups.

### Evaluation of Disease Activity of Dextran Sulfate Sodium-Induced Colitis

At the same time, the life status, fecal condition (fecal morphology and occult blood phenomenon), and body weight of mice were assessed every day. The disease activity index (DAI) was determined by scoring changes in body weight, blood in stool, and stool consistency, as described previously. Briefly, we used five grades of weight loss (no loss = 0, 1–5% = 1, 5–10% = 2, 10–15% = 3, >15% = 4), five grades of stool consistency (normal = 0, mild looseness = 1, looseness = 2, diarrhea = 3, bloody stool = 4), and three grades of occult blood condition (negative = 0, positive = 2, bloody stools visible to the naked eye = 4). The combined scores were then divided by three to obtain the final DAI scores.

### Induction and Treatment of Azoxymethane/Dextran Sulfate Sodium-Induced Colorectal Cancer

The azoxymethane/dextran sulfate sodium (AOM/DSS)-induced CRC model has been described previously (Liang et al., 2013). As shown in Figure 4A, AOM (Sigma-Aldrich, Hamburg, Germany) was administered by intraperitoneal injection at 10.0 mg per kg body weight (mg/kg). After 7 days, mice received 2.0% DSS in autoclaved water for 7 days followed by recovery on sterile water for 14 days. Three DSS cycles were performed. Oral administration with *B. longum* or *B. longum-Endo* was performed for 14 days and stopped for 7 days, with four cycles in total. The grouping was the same as described in DSS-induced colitis. To better monitor disease progress, we dissected two mice per group randomly every month. All mice were dissected 15 weeks after the first injection of AOM.

### Hematoxylin and Eosin Staining and Histological Score

Formalin-fixed and paraffin-embedded colorectum tissue was cut in 4 μm sections and stained according to the standard protocol of hematoxylin and eosin (H&E) staining assay. The histological score was evaluated as described previously (Vieira et al., 2009). Briefly, we assessed the severity of inflammation (none = 0, mild = 1, moderate = 2, severe = 3), depth of injury (no mucosal injury = 0, mucosal injury = 1, mucosa and submucosal injury = 2, injury across the intestinal wall = 3), and inflammatory cell infiltration (no or very few inflammatory cells in the lamina propria of the mucosa = 0, more inflammatory cells in the lamina propria of the mucosa = 1, inflammatory cells spread to the submucosa = 2, exudation of inflammatory cells throughout the layer = 3). The combined scores were the colorectal histology score with a total scoring range of 0–9 per mouse.

### RNA Extraction and Quantitative Real-Time PCR

According to the manufacturer’s instructions, the total RNA of colonic tissue was extracted using Trizol reagent (Vazyme, Nanjing, China). The RNA concentration and quality were determined using a microplate reader for each sample by the 260/280 nm ratio. RNA was reversely transcribed into cDNA using a ReverTra Ace™ qPCR RT Kit (TOYOBO, Japan) according to the manufacturer’s instructions. Quantitative real-time PCR (qRT-PCR) was performed using an AceQ^®^ qPCR SYBR Green Master Mix (Vazyme, Nanjing, China) and real-time PCR amplification system (Thermo Fisher Scientific, Waltham, MA, United States). The primer sequences were listed in Supplementary Table 1. Each qRT-PCR reaction was repeated at least three times and β-actin was used as an internal control.

### 16S rRNA Gene Sequencing

The fecal samples of per mouse were collected in 1.5 mL tubes and immediately frozen in liquid nitrogen. In this study, the number of animals in “Control” was 6, and other treatment groups were all 8. Before sequencing, we mixed mice fecal samples of two mice per group and then extracted DNA. 16S rRNA gene sequencing was performed as described previously in detail (Caporaso et al., 2011). Briefly, genomic DNA was extracted using a QIAamp DNA Stool Mini Kit (51504, QIAGEN) according to the manufacturer’s instructions. The V3–V4 variable regions of the bacterial 16S rRNA gene were amplified by PCR with specific primers: 338F (5′-ACTCCTACGGGAGGCAGCAG-3′) and 806R (5′-GGACTACHVGGGTWTCTAAT-3′). The PCR condition was as follows: initial denaturation at 94°C for 4 min, followed by 25 cycles of 94°C denaturation for 30 s, 50°C annealing for 45 s, and 72°C extension for 30 s; final extension at 72°C for 5 min. PCR products were cleaned and subsequently sequenced by an Illumina MiSeq PE300 system (OEbiotech Co, Ltd.) according to the standard protocols. The resultant sequences were screened for quality using the QIIME software package (version 1.9.1) and the effective sequences were further clustered into Operational Taxonomic Units (OTUs) of ≥97% similarity. The relative abundance of each OTUs, other taxonomic levels (from phylum to genus), beta-diversity (between-sample dissimilarity), and principal component analysis (PCA) were performed for each sample by using MOTHUR program.

### Functional Annotation and Prediction of Microbiota

The microbial function was predicted using PICRUSt based on the abundance at the OTU level (Langille et al., 2013). The OTUs were mapped to the gg13.5 database at 97% similarity by QIIME’s command ‘‘pick_closed_otus.’’ Then, OTU abundance was normalized automatically using 16S rRNA gene copy numbers from known bacterial genomes of the Integrated Microbial Genomes^1^ database. The predicted genes and their function were annotated with the Kyoto Encyclopedia of Genes and Genomes (KEGG)^2^ database, and the differences between groups were compared using the online platform STAMP^3^ (Parks and Beiko, 2010). A two-sided Welch’s *t*-test and Benjamini–Hochberg false-discovery rate (*P* < 0.05) correction were used in the two-group analysis.

### Statistical Analysis

Experiments were performed at least three times with similar results. The results were presented as the mean ± standard deviation (SD) after the analyses were completed with Graph Pad Prism 8.0 (Graph Pad Software, San Diego, CA, United States). Comparisons between two experimental groups were performed using a two-tailed Student’s *t*-tests. For multiple comparisons, data were analyzed using a one-way analysis of variance with a Tukey post-test. For all analyses, *P* < 0.05 was considered statistically significant.

## Results

### Cloning and Expression of Endostatin in *Bifidobacterium longum*

**FIGURE 1:**
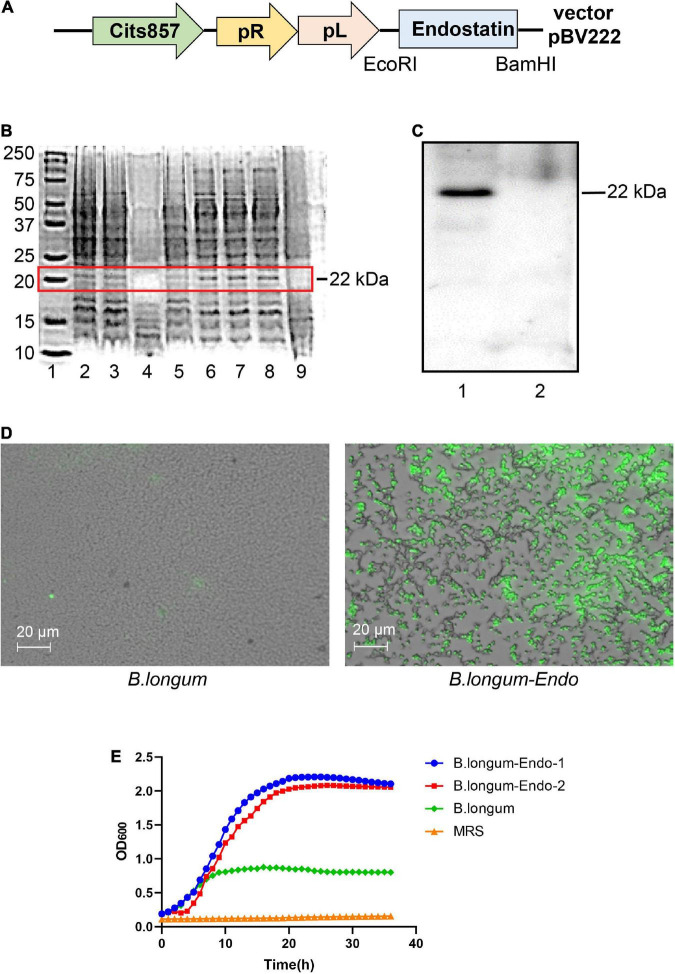
Cloning and expression of endostatin in *Bifidobacterium longum*. **(A)** Schematic representation of expression cassettes for controlled and targeted endostatin production in *B. longum*. **(B)** Expression of endostatin in probiotic strains. Lane 1: Protein marker; Lanes 2–3: *L. lactis-Endo*; Lane 4: *L. lactis*; Lane 5: *L. acidophilus*; Lane 6: *L. acidophilus-Endo*; Lanes 7–8: *B. longum-Endo*; and Lane 9: *B. longum*. **(C)** Expression of endostatin in *B. longum*. Lane 1: *B. longum-Endo*; Lane 2: *B. longum.* **(D)** Detection of endostatin protein expression in *B. longum* by immunofluorescence staining. **(E)** The growth curve of the recombinant strain was measured with the wild strain and MRS solution as control.

The whole endostatin gene of 560 bp was amplified from cDNA of human placenta and cloned into the pPRPL-induction vector pBV222 (Figure 1A). The resulting recombinant plasmids pBV222 and pBV222-endostatin were transformed into *Lactococcus lactis NZ9000*, *Lactobacillus acidophilus LMG P-219043*, and *B. longum* ATCC 55813 (*B. longum*), respectively. After induction at 42°C for 8 h, total cell protein and secreted protein in the culture supernatant were separately extracted. As shown in Figure 1B, the expected 22 kDa protein product was successfully expressed in three recombinant strains. Among these recombinant strains, *B. longum* showed the highest relative expression of endostatin. We confirmed this result using western blotting and immunofluorescence staining. As shown in Figure 1C, the strain *B. longum* expressed a high level of the endostatin protein (designated as *B. longum-Endo*); however, no endostatin band was found in the parent strain harboring the empty vector pBV222 (designated as *B. longum*). Similarly, no fluorescence was observed in the wild-type strain, while the recombinant strain carried an obvious fluorescence signal (Figure 1D). These results suggested that endostatin protein was successfully expressed in *B. longum*. Surprisingly, the newly constructed recombinant strains displayed an accelerated growth rate. As shown in Figure 1E, the OD value of the wild-type strain was only 0.823 at 24 h, while those of the two recombinant strains were 2.207 and 2.07, respectively. We hypothesized that the recombinant strain *B. longum-Endo* has a higher growth rate, which provides the advantage for performing biological functions *in vivo*.

### *Bifidobacterium longum* Alleviates Dextran Sulfate Sodium-Induced Acute Inflammatory Bowel Disease in Mice

**FIGURE 2:**
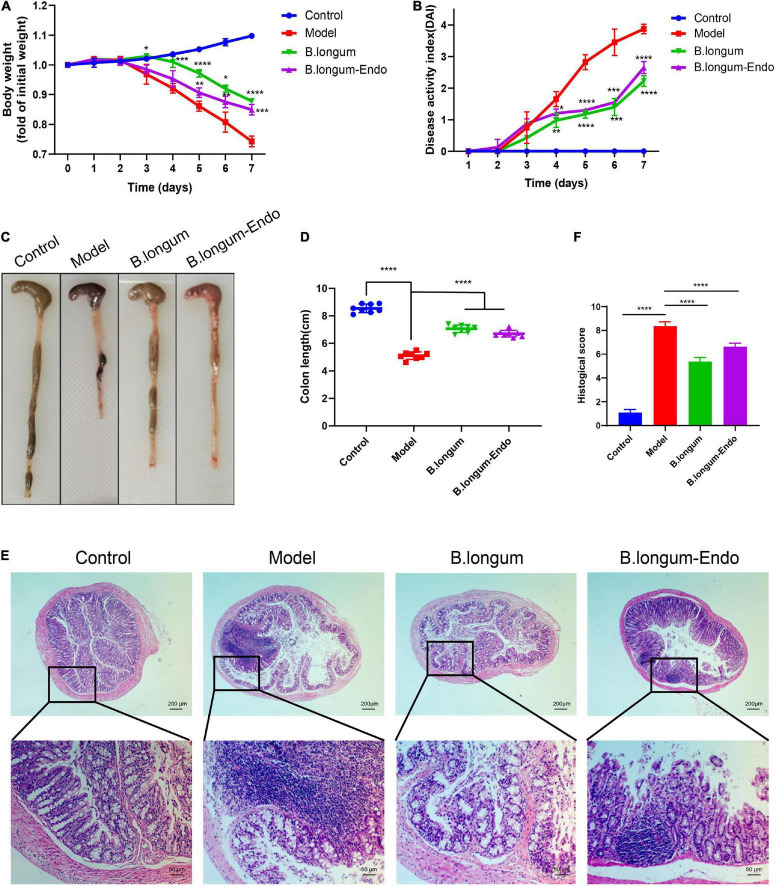
*B. longum* alleviates DSS-induced acute inflammatory bowel disease in mice. **(A)** Percentage change in body weight. **(B)** Disease activity index score, a composite measure of weight loss, stool consistency, and blood in stool. **(C)** Representative photographs showing colon tissue. **(D)** Changes in colon length. **(E)** Representative hematoxylin and eosin-stained sections of colon. **(F)** Histopathological scores. Data are represented as mean ± SD of three independent experiments (*n* = 8). *P < 0.05, **P < 0.01, ***P < 0.001, ****P < 0.0001, and ns: not significant.

We firstly established an acute colitis model in the current study by treating mice with 3% DSS. One day after treatment, mice were administered with normal saline (200 μL/per mouse), *B. longum* [1.5 × 10^10^ colony-forming units (CFU)/kg], or *B. lon*gum*-Endo* (1.5 × 10^10^ CFU/kg). During this process, body weight, stool consistency, and fecal occult blood were monitored daily, and each mouse’s DAI was further scored. As shown in Figure 2A, the body weight in the control group increased gradually, whereas that of all the treatment groups with DSS decreased considerably as time elapsed. Meanwhile, we found that *B. longum* and *B. longum-Endo* significantly slowed down the body weight decrease of mice in the model group. The body weight of mice in the untreated model group decreased to 77% of the starting weight on the seventh day, while that for *B. longum* and *B. longum-Endo* groups were 87.8 and 84.9%, respectively. Moreover, *B. longum* and *B. longunm-Endo* groups showed a significantly lower stool consistency score and fecal bleeding index. Their final DAI score was reduced by 42.5 and 31.6% compared with the model mice (*P* < 0.0001; Figure 2B). When mice were dissected, there were almost no well-formed stools fecal of colonic tissue in the untreated model group, suggesting that the DSS-induced mouse model had a severe colitis symptom (Figure 2C). Correspondingly, colon length of the model group was also significantly shortened (Figure 2D). In contrast, *B. longum-* and *B. longum-Endo*-treated mice exhibited more formed stool as well as an increase in colon length, indicating that the pathological changes induced by DSS were alleviated after treatment. Furthermore, H&E staining of the colonic tissue showed less reduced villus length, disrupted crypt architecture, and fewer signs of inflammation (Figures 2E,F). After administration of *B. longum* or *B. lon*gum-*Endo*, no morphological alterations were evident in close examinations of multiple organs including liver, spleen, lung, and kidney (Supplementary Table 2 and Supplementary Figure 2). Altogether, these results indicated that both *B. longum* and *B. lon*gum*-Endo* improved DSS-induced acute IBD in mice.

### *Bifidobacterium longum-Endo* Administration Further Accelerates the Recovery of Dextran Sulfate Sodium-Induced Acute Inflammatory Bowel Disease in Mice

**FIGURE 3:**
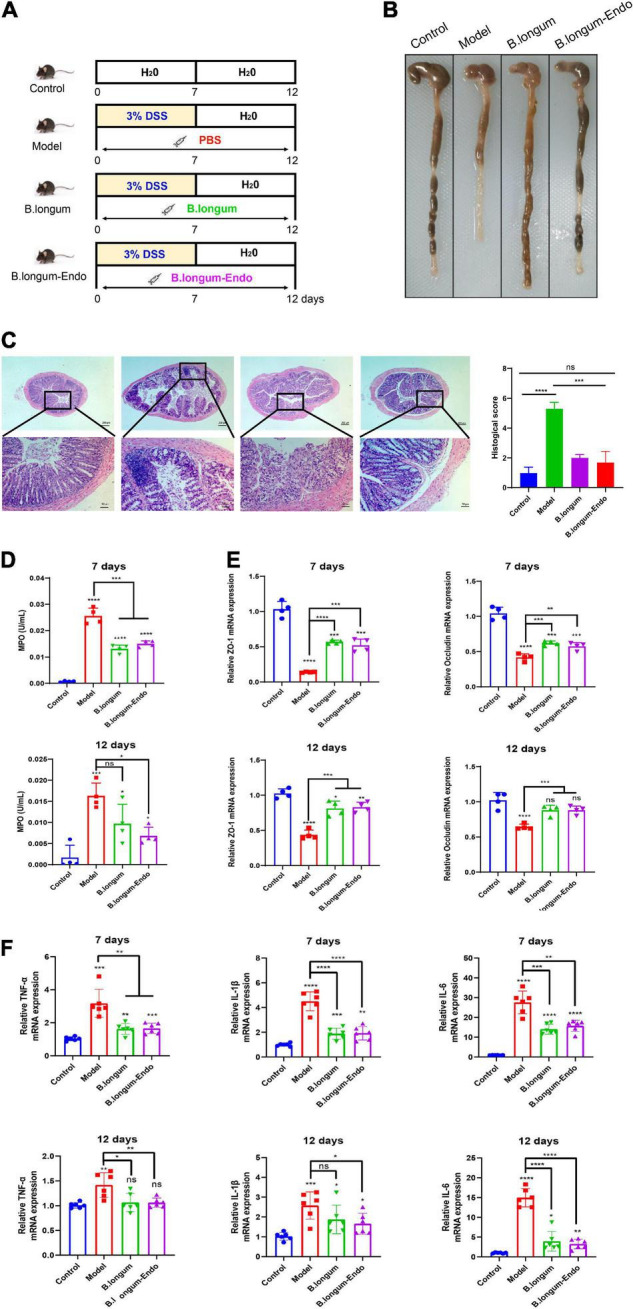
*B. longum-Endo* administration further accelerates the recovery of DSS-induced acute inflammatory bowel disease in mice. **(A)** Schematic diagram of rehabilitation experimental model of acute colitis. The mice drank water normally for 5 days after taking 3% DSS for 7 days. **(B)** Representative photographs showing colon tissue. **(C)** Representative hematoxylin and eosin-stained sections of colon and histopathological scores. **(D)** Detection of myeloperoxidase (MPO) activity at days 7 and 12. **(E)** Detection of tight junction proteins ZO-1 and Occludin expression levels by qRT-PCR analysis at days 7 and 12. **(F)** Detection of proinflammatory cytokines TNF-α, IL-1β, and IL-6 expression levels by RT-PCR analysis at days 7 and 12. Data are expressed as mean ± SD of three independent experiments (*n* = 6–8). *P < 0.05, **P < 0.01, ***P < 0.001, ****P < 0.0001, and ns: not significant.

To further investigate the beneficial effects of *B. longum-Endo* on DSS-induced colitis, we performed a recovery experiment of acute colitis. As shown in Figure 3A, the mice were administered 3.0% DSS for 7 days to cause acute epithelial injury, followed by 5 days of normal drinking water to allow for repair. We find that body weight losses were evident in mice treated with DSS; however, mice could regain their body weight after administration with normal drinking water (Supplementary Figure 3A). Compared to the model group, oral administration of *B. longum* or *B. longum-Endo* significantly decreased inflammation severity, as evidenced by the DAI scores (Supplementary Figure 3B). Surprisingly, the mean DAI score of the *B. longum-Endo* group was only 0.43 on the 12th day, suggesting that inflammation was almost completely recovered. As shown in Figure 3B, mice with *B. longum-Endo* treatment exhibited a better fecal consistency and firmer stool than the *B. longum* group. Pathology scoring of H&E-stained colon tissue sections harvested on the 12th day also indicated that *B. longum-Endo* treatment had a better therapeutic effect (Figure 3C). Myeloperoxidase (MPO) activity can be used as a marker of inflammation. In this study, we found that MPO activity of mice showed a significant increase after DSS administration for 7 days, whereas *B. longum* and *B. longum-Endo* treatment reduced the MPO level in colon tissues. Notably, compared with the untreated model group, the *B. longum-Endo* group showed a lower MPO on the 12th day, whereas no significant difference was detected in the *B. longum*-treated group (Figure 3D). In addition, the expression levels of ZO-1 and Occludin were significantly decreased in the DSS-induced group, while *B. longum-Endo* treatment effectively alleviated such changes (Figure 3E). Furthermore, real-time RT-PCR analysis was performed to assess the expression of TNF-α, IL-1β, and IL-6. The results showed that gene expression levels of pro-inflammatory factors were significantly upregulated in the model group. However, on the 12th day following *B. longum-Endo* treatment, the expression of TNF-α, IL-1β, and IL-6 were significantly downregulated, although not to that of the control (Figure 3F). Overall, oral delivery of the *B. longum-Endo* showed a robust conclusion on the therapeutic effect of IBD.

### *Bifidobacterium longum-Endo* Inhibits Azoxymethane/Dextran Sulfate Sodium-Induced Colon Tumorigenesis

**FIGURE 4:**
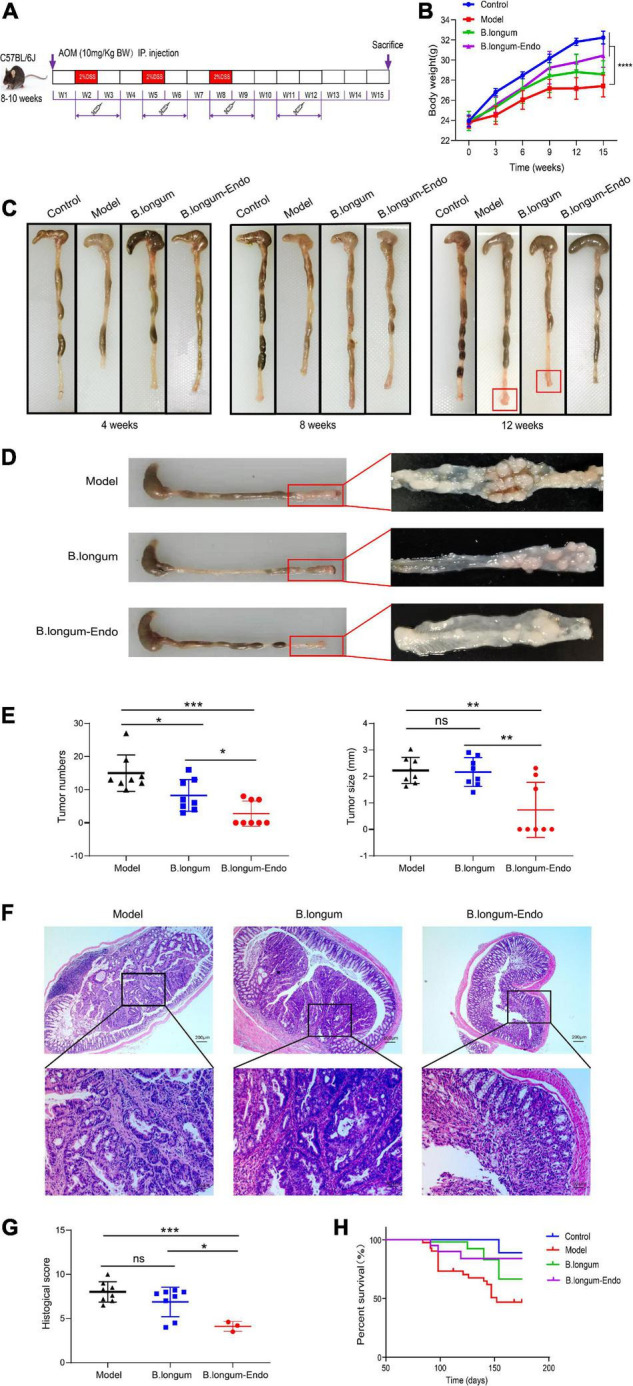
*B. longum-Endo* inhibits AOM/DSS-induced colon tumorigenesis. **(A)** Schematic representation of AOM/DSS-induced colitis-associated colon cancer mouse model. **(B)** Body weight. **(C)** Representative photographs showing colon tissue at different stages. **(D)** The formation of solid tumors in the distal colon was observed by naked eye. **(E)** Tumor number and tumor size. **(F,G)** Representative hematoxylin and eosin-stained images and histopathological scores. **(H)** The 25-week survival curve after three 2% DSS cycles. Data are expressed as mean ± SD of three independent experiments (*n* = 6–8). *P < 0.05, **P < 0.01, ***P < 0.001, ****P < 0.0001, and ns: not significant.

Colorectal cancer is a chronic inflammatory disorder, and various inflammation mediators control its initiation and progression. To further examine whether *B. longum-Endo* could be used to treat or delay pathological processes of CRC, we performed an AOM/DSS-induced colitis-associated colon cancer mouse model (Figure 4A). As shown in Figure 4B, AOM/DSS treatment significantly decreased body weight of mice, while *B. longum-Endo* alleviated this change. The body weight of the *B. longum-Endo* group was increased by approximately 11.08% compared to that of the model group. We dissected two mice per group randomly every month to assess their overall status during the experiment period. Multiple parameters, including body weight, spleen weight, and colon morphology, showed that *B. longum-Endo* administration alleviated chronic inflammation caused by AOM/DSS treatment. Interestingly, we found that small intestinal length displayed a similar change trend to the colon length (Tables 1–3). On the 12th week, one mouse in the model and *B. longum* groups developed macroscopic tumor nodules (Figure 4C). All mice were sacrificed 15 weeks later, and we observed that all eight mice of the model and *B. longum* groups developed grossly visible tumors at the distal colon or rectum. Notably, only three out of the eight mice developed tumors in the *B. longum-Endo* group. Compared to the wild *B. longum* therapy, recombinant *B. longum-Endo* showed a better antitumor effect *in vivo*. As shown in Figures 4D,E, the colon tumor numbers and tumor size were remarkably lower in *B. longum-Endo-*treated mice than in *B. longum-*treated mice (*P* < 0.05). Furthermore, immunohistochemistry analysis of tumor tissues showed that the disease score of the *B. longum-Endo* group was significantly lower than that of the model and *B. longum* groups (Figures 4F,G). Meanwhile, we found that oral *B. longum-Endo* administration also significantly prolonged the survival rate of mice (Figure 4H). Taken together, these data demonstrated that *B. longum-Endo* treatment potently suppressed colorectal tumorigenesis.

**TABLE 1:** Organ parameter changes in mice at 4 weeks after AOM injection.

4 weeks						
Group	Body weight (g)	Liver (g)	Spleen (g)	Kidney (g)	Small intestine length (cm)	Colon length (cm)
Control-1	26.9	1.426	0.068	0.313	39	8.59
Control-2	27.7	1.424	0.072	0.327	37.3	8.13
Model-1	24.8	1.314	0.670	0.298	33.1	6.2
Model-2	25.3	1.369	0.683	0.293	35.2	6.36
*B. longum*-1	24.3	1.268	0.680	0.282	37.4	7.36
*B. longum*-2	26.0	1.386	0.702	0.494	36.2	7.05
*B. longum*-Endo-1	26.7	1.410	0.721	0.320	37.2	7.42
*B. longum*-Endo-2	26.0	1.420	0.705	0.312	35.5	7.78

**TABLE 2:** Organ parameter changes in mice at 8 weeks after AOM injection.

8 weeks						
Group	Body weight (g)	Liver (g)	Spleen (g)	Kidney (g)	Small intestine length (cm)	Colon length (cm)
Control-1	29.8	1.582	0.775	0.334	36.6	7.89
Control-2	28.2	1.531	0.705	0.327	35	8.20
Model-1	26.2	1.420	0.760	0.304	32.4	7.49
Model-2	27.1	1.507	7.859	0.320	33.5	7.32
*B. longum*-1	27.1	1.480	0.786	0.325	35.7	8.12
*B. longum*-2	29.9	1.555	0.852	0.359	36.8	8.31
*B. longum*-Endo-1	27.3	1.502	0.764	0.322	34.3	7.74
*B. longum*-Endo-2	30.6	1.655	0.857	0.355	33.2	7.26

**TABLE 3:** Organ parameter changes in mice at 12 weeks after AOM injection.

12 weeks						
Group	Body weight (g)	Liver (g)	Spleen (g)	Kidney (g)	Small intestine length (cm)	Colon length (cm)
Control-1	31.800	1.654	0.789	0.347	35.4	8.49
Control-2	33.500	1.782	0.908	0.395	35.6	8.10
Model-1	26.800	1.514	0.911	0.319	30.8	7.82
Model-2	27.500	1.491	0.963	0.344	32	7.98
*B. longum*-1	27.800	1.562	0.834	0.334	33.4	7.74
*B. longum*-2	28.400	1.500	0.852	0.349	34.6	7.52
*B. longum*-Endo-1	28.500	1.519	0.827	0.342	35	7.49
*B. longum*-Endo-2	29.900	1.588	0.867	0.362	32.8	7.21

### *Bifidobacterium longum-Endo* Improved Intestinal Flora Dysbiosis Caused by Azoxymethane/Dextran Sulfate Sodium

**FIGURE 5:**
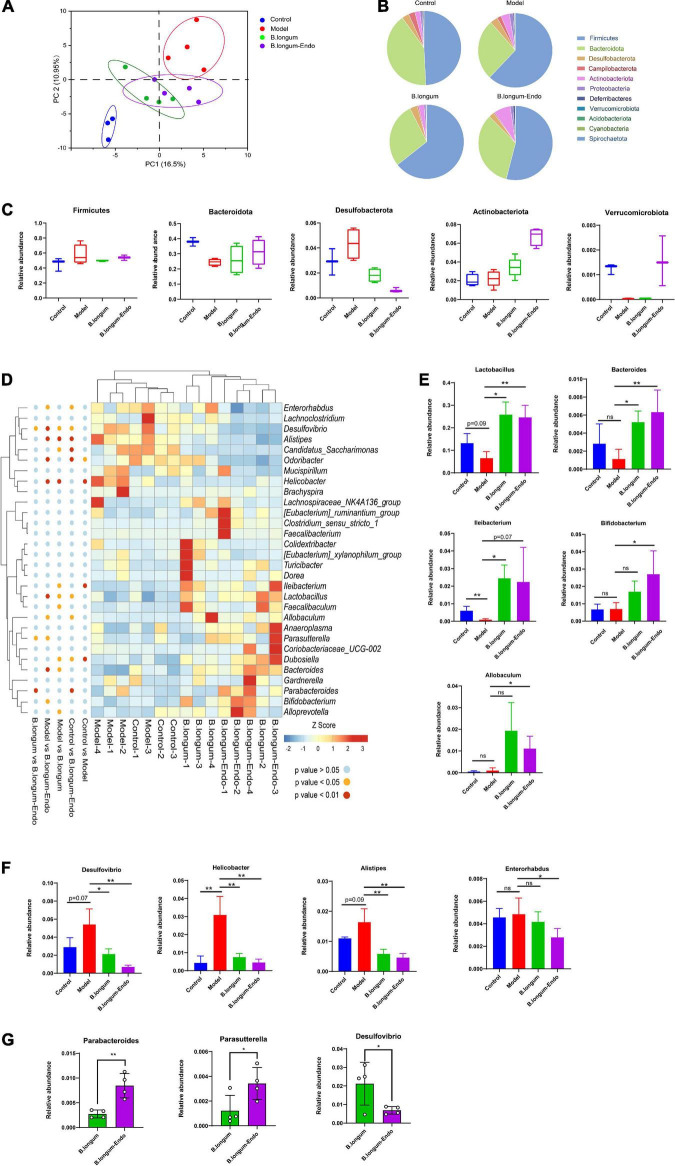
*B. longum-Endo* improved intestinal flora dysbiosis caused by AOM/DSS. **(A)** Principal components analysis (PCA). **(B)** Overview of relative abundance of microbiota at phylum level in mice. **(C)** The relative abundance changes of *Firmicutes*, *Bacteroidota*, *Desulfobacterota*, *Actinobacteria*, and *Verrucomicrobiota* after *B. longum-Endo* treatment. **(D)** Heatmap depicting the relative abundance of bacterial species at genus level for each mouse. **(E)** Relative abundance of representative beneficial bacteria (*Lactobacillus*, *Bacteroides*, *Ileibacterium Bifidobacterium*, and *Allobaculum*). **(F)** Relative abundance of representative harmful bacteria (*Desulfovibrio*, *Helicobacter*, *Alistipes*, and *Enterorhabdus*). **(G)** Representative differential microbiota (*B. longum-Endo* group vs. *B. longum* group, *P* < 0.05). *P < 0.05; **P < 0.01; ns: not significant.

Accumulating evidence suggests that intestinal microorganisms are associated with the development of CRC. To determine whether *B. longum-Endo* treatment affected the gut microbiota, we thus performed 16S ribosomal (rRNA) gene sequencing of fecal samples from the AOM/DSS-treated mice. PCA showed a clear separation between the model and *B. longum-Endo* groups, suggesting that *B. longum-Endo* intervention did alter the gut microbial composition (Figure 5A). Based on the species profile, we calculated the alpha diversity to estimate gut microbiota community richness and microbial diversity. As shown in Supplementary Figure 4, the ACE, Chao1, and Shannon’s indexes of alpha diversity increased significantly in the *B. longum-Endo* group. However, no significant difference in Simpson’s index was found among the four groups. The gut microbiota community distribution and relative abundance of taxa were further analyzed (Figure 5B). We found that the gut microbiota of all mice was dominated by *Firmicutes* and *Bacteroidota*, accounting for more than 80% of all bacterial flora. After induction with AOM/DSS, the relative abundance of *Firmicutes* was significantly increased compared with the control group. Compared with the model group, the relative *Bacteroidota* abundance in the *B. longum-Endo* treatment group increased by approximately 27.19%, while the *Desulfobacterota* abundance decreased by 48.44%. Strikingly, the relative abundance of *Actinobacteriota* phylum was almost twice higher in the *B. longum-Endo* group than that in the model group (Figure 5C). In addition, we found that the relative abundance of *Verrucomicrobiota* was significantly decreased in the model group, while *B. longum-Endo* treatment reversed the alteration. Notably, the administration of wild *B. longum* did not improve *Verrucomicrobiota* abundance. Next, the changes of genus level in fecal microbiota composition were further analyzed. Here, we focused on the top 30 intestinal microflora, and the results were presented in Figure 5D. Although the individual difference was observed, the main gut microbiota diversity changes showed a consistent trend. AOM/DSS treatment reduced the abundance of beneficial bacteria and increased potential harmful bacteria. As expected, following *B. longum* and *B. longum-Endo* treatment, the imbalanced structure of the intestinal flora was improved. As shown in Figure 5E, the abundance of *Lactobacillus* was 24.6% in the *B. longum-Endo* group but only 9.29% in the model group. Similarly, *Bacteroides* and *Ileibacterium* abundance showed an increasing trend in the *B. longum-Endo* group. Notably, *B. longum-Endo* treatment significantly increased the abundance of *Bifidobacterium* and *Allobaculum*, while no significant differences were observed between the model and *B. longum* groups (Figure 5E). Furthermore, we found that *B. longum-Endo* decreased the relative abundance of potentially pathogenic genera, such as *Desulfovibrio*, *Helicobacter*, *Alistipes*, and *Enterorhabdus* (Figure 5F). Compared with the *B. longum* group, the relative abundance of *Desulfovibrio* was significantly decreased (*P* < 0.05) in the *B. longum-Endo* group, while *Parabateroides* and *Parasutterella* abundance were significantly increased (Figure 5G). Altogether, these results suggested that *B. longum-Endo* improved the intestinal flora dysbiosis caused by AOM/DSS.

### Predictive Functional Profiles of Bacterial Communities

To determine the significant pathways involved in differential bacteria, we used the KEGG pathway database to predict putative functions. Table 4 presented the predicted microbial functions at level 1 of the KEGG pathways. After AOM/DSS treatment, four functional orthologs including cellular processes, organismal systems, environmental information processing, and human diseases were significantly altered. Compared with the model group, the abundance of bacteria involved in metabolism functional ortholog was significantly decreased in the *B. longum* group. In contrast, the abundance of bacteria related to environmental information processing was significantly decreased in the *B. longum-Endo* group. A heat map reflecting hierarchical clustering of relative bacterial abundances and their associated function orthologs in level 2 and 3 KEGG pathways were presented in Supplementary Figure 5. In level 2 KEGG pathways, intestinal flora with high abundance in the *B. longum-Endo* group was related to pathways including cancers, endocrine_and_metabolic_disease, folding_sorting_and_degradation, and translation (Supplementary Figure 5A). Furthermore, differential bacteria (*P* < 0.05, Model vs. *B. longum-Endo* or Model vs. *B. longum*) were analyzed based on the third-level KEGG pathways (Supplementary Figure 5B). Compared with the model group, the increased microbiota in the *B. longum-Endo* group codifies functions in KEGG pathways related to prokaryotic defense system, sulfur relay system, steroid hormone biosynthesis, protein digestion and absorption, CD molecules, small cell lung cancer, systemic lupus erythematosus. Meanwhile, we found that the decreased microbiota in the *B. longum-Endo* group (*P* < 0.05) were related to 23 pathways. Among these, significant differential bacteria (*B. longum-Endo* vs. Model, *P* < 0.01), such as *Desulfovibrio*, *Helicobacter*, and *Alistipes*, were related to oxidative phosphorylation, renin-angiotensin system and apoptosis pathways. Here, we found that two pathways, namely oxidative phosphorylation and lysine biosynthesis, showed significant differences between the *B. longum* group and the *B. longum-Endo* group.

**TABLE 4:** Predicted KEGG functional pathway differences at the first level using PICRUSt.

KO functions	Relative abundance%				*P*-value			
Level 1	Control	Model	*B. longum*	*B. longum*-Endo	Control vs. Model	Model vs. *B. longum*	Model vs. *B. longum*-Endo	*B. longum* vs. *B. longum*-Endo
Cellular Processes	8.14	7.19	7.00	6.88	0.08	0.70	0.60	0.84
Organismal systems	1.76	1.54	1.47	1.51	0.02	0.43	0.65	0.70
Environmental information processing	13.81	14.28	14.26	14.02	0.001	0.88	0.05	0.15
Human diseases	2.77	2.96	2.94	2.91	0.06	0.90	0.60	0.70
Metabolism	43.07	42.83	42.25	42.29	0.38	0.05	0.20	0.92
Genetic information processing	24.5	25.5	26.34	26.75	0.12	0.14	0.22	0.67

## Discussion

Probiotics have been increasingly applied in the food and pharmaceutical industry to prevent disease and improve host health. Some studies have demonstrated that oral delivery of specific probiotic strains such as *Bifidobacterium* could improve intestinal immunity and alleviate IBD and CRC. In the current study, we constructed a recombinant strain of *B. longum-Endo*, which expressed a high level of endostatin protein, and demonstrated its ability of anti-inflammation and anti-tumor in animal models for the first time.

*Bifidobacterium* as a therapeutic delivery system has many advantages: (1) *Bifidobacterium* itself has a variety of physiological functions, such as anti-inflammation and anti-tumor effects. (2) *Bifidobacterium* exhibits a strong colonization ability. It appears in the gut shortly after birth and can be detected throughout an individual’s lifespan. Compared to the *Lactobacillus*, *Bifidobacterium* is more accessible to be colonized in the gut, which is very suitable for expressing some peptide drugs in the intestinal tract (Drolia et al., 2020; Miranda et al., 2021). (3) Good safety. The body has immune tolerance to *Bifidobacterium* since its long-term colonization in the intestine. Therefore, long-term use will not cause rejection. (4) *Bifidobacterium* displays superior *in vivo* tumor-targeting ability. When genetically engineered *Bifidobacterium* was introduced systemically into tumor-bearing mice, bacteria were found only in the tumors, presumably due to the hypoxic environment required for the growth of these bacteria (Yazawa et al., 2000). Despite the above merits, the research progress on *B. longum* is far from satisfactory. Indeed, the “probiotic-based delivery system” genetic engineering is still in its infancy. As a Gram-positive cell, *Bifidobacterium* spp. has thick cell walls and needs a strictly anaerobic environment, which undoubtedly increases the technical difficulty of operation (Hong et al., 2021). To our knowledge, the genetic transformation technique in *B. longum* is still relatively immature, and commercialized carrier of *B. longum* is limited. To overcome these difficulties, new strategies have been explored.

Our previous study has successfully constructed recombinant strains *L. lactis-Endo*, *L. acidophilus-Endo*, and *B. longum-Endo*, and the expression of endostatin was the highest in *B. longum*. In this study, we further explored the effect of *B. longum-Endo* on the treatment of IBD in animal models. The results indicated that oral administration of *B. longum-Endo* attenuated DSS-induced acute colitis, as demonstrated by a reduction of colitis indices, intestinal shortening, and histological changes at both macroscopic and microscopic levels. Proinflammatory factors are closely related to intestinal inflammation and clinical symptoms of IBD. TNF-α, a canonical pro-inflammatory cytokine, was found to be upregulated in colon tissue of IBD patients (Murch et al., 1993). In the animal models, including TNBS and DSS-induced colitis, TNF-α was also significantly upregulated (Marini et al., 2003). Herein, we demonstrated that *B. longum-Endo* reduced the level of TNF-α at the inflammation site, and by 12 days after treatment, TNF-α expression returned to the basal level. Early studies implicated that IL-6 is a multifunctional cytokine produced during inflammation; inhibiting the IL-6/STAT3 signaling pathway may be a potential therapeutic target for IBD (Matsumoto et al., 2009). Notably, IL-6 was the most responsive to *B. longum-Endo* in treating colitis, with its expression level changed by 4.6 times after treatment. As a marker of tissue damage and neutrophil infiltration, MPO activity is closely correlated with IBD severity (Zhao et al., 2019). MPO levels are usually upregulated in IBD patients (Ma et al., 2004). In agreement with previous studies, we also detected high levels of MPO in the colon tissue of mice induced by DSS, and *B. longum-Endo* reduced MPO activity significantly. Collectively, recombinant *B. longum-Endo* achieved therapeutic effect without toxic side effects on colitis.

Chronic inflammation, one of the “promoting forces” in the tumor microenvironment, has been suggested to be associated with the initiation, promotion, and progression of tumorigenesis. Recent prospective observational studies suggested that IBD patients displayed an increased prevalence of CRC (Gillen et al., 1994; Derikx et al., 2017; Samadder et al., 2019; Weimers et al., 2021). In this study, we constructed an AOM/DSS mouse model by which chronic inflammation is a major driver of tumorigenesis and promotion. Our data showed that *B. longum-Endo* played a critical role in tumorigenesis and promotion, with evidence that the disease score and tumor formation rate were lower than that of the untreated model group and *B. longum* group. Compared with the wide *B. longum*, recombinant *B. longum-Endo* showed superior anti-tumor efficacy with lower tumor numbers and tumor size, which seems to be well accounted for by the endostatin protein. Endostatin is a 20–28 kDa proteolytic fragment of collagen XVIII with potent antiangiogenic activity (O’Reilly et al., 1997). Recombinant endostatin has been considered as a broad-spectrum anticancer agent because of its endogenous inhibitory effect on angiogenesis, non-toxicity, and synergistic ability with chemotherapy drugs (Boehm et al., 1997; Folkman, 2006; Zhuang and Yuan, 2009). Currently, recombinant endostatin (Endostar) has been approved for the treatment of NSCLC by the State Food and Drug Administration of China, which is expressed and purified in *E. coli* with an additional nine-amino acid sequence (Hu et al., 2014). However, its short half-life and instability limit clinical applications. Great efforts are currently devoted to gene therapy by which endostatin can be delivered and expressed in specific tumor regions *in vivo* (Li et al., 2003; Ma et al., 2004). Jia et al. (2004) reported that endostatin slowed or even stopped tumor growth, but when treatment stopped, tumors began to re-grow rapidly. Pleasingly, we found that *B. longum* as a delivery system to carry endostatin showed long-term safety and efficacy for colon cancer. When administration with *B. longum-Endo* stopped, the primary tumor did not grow, and the mouse’s overall survival rate was also improved. We thus concluded that the combination of *B. longum* and endostatin showed self-amplifying, synergistic anti-inflammation, and anti-tumor effects in CRC.

Increasing evidence has indicated that gut microbiota plays an essential role in maintaining intestinal homeostasis (Marchesi et al., 2016). Dysbiosis of the gut microbiota is closely related to IBD and CRC development (Peterson et al., 2015). For example, decreased beneficial *Lactobacillus* and increased potential harmful *Enterobacteria* are closely related to IBD severity and treatment effectiveness (Zhou et al., 2018). Our study revealed that the relative abundance of potentially pathogenic bacteria including *Helicobacter*, *Desulfovibrio*, *Alistipe*s, and *Enterorhabdus* increased in AOM/DSS-induced mice, but decreased after *B. longum-Endo* treatment. *Desulfovibrio* is a genus of sulfate-reducing bacteria that is ubiquitous in oligotrophic and eutrophic environments. Overproduction of H_2_S by *Desulfovibrio* in the colon has been implicated in colonic inflammation and cancer (Loubinoux et al., 2002; Carbonero et al., 2012). Compared to the wild *B. longum*, *B. longum-Endo* treatment significantly decreased the relative abundance of *Desulfovibrio*, which may contribute to inhibiting H_2_S generation. Notably, *Alistipe*s, one of the top ten most abundant genera associated with human colorectal carcinomas, has been proposed as a potential biomarker for CRC (Moschen et al., 2016). Oral supplementary of recombinant *B. longum-Endo* heightened colonization of *Bifidobacterium* in the gut and increased proliferation of *Bifidobacterium* species. In this study, we found that the relative abundance of intestinal *Bifidobacterium* was significantly increased only in the *B. longum-Endo* group. Among the taxa identified, the most notable were the *Parabacteroides* and *Parasutterella* in the *B. longum-Endo* group. *Parabacteroides* is a core component of the human and mouse gut microbiota and has been correlated with various health outcomes. Administration of *Parabacteroides* alleviated heparanase-exacerbated acute pancreatitis through reducing neutrophil infiltration (Lei et al., 2021). In accordance with our study, several studies recently reported that *Pabacteroides* alleviated AOM/DSS-induced inflammation (Koh et al., 2018; Koh et al., 2020). *Parasutterella* is still a relatively new genus with limited literature. Henneke et al. (2022) reported *Parasutterella* was associated with obesity and type 2 diabetes, but not with systemic inflammatory markers like IL-6. Interestingly, IL-6 reflects the degree of metabolic inflammation in obesity (Park et al., 2005). In IBS patients, it has been observed that altered *Parasutterella* is commonly linked with changes in gastrointestinal function (Chen et al., 2018). Our result indicated that *B. longum-Endo* significantly increased the abundance of *Parasutterella* in the gut, which might be an attractive candidate for future investigations. In addition to the effects of *B. longum-Endo* on cancer development inhibition, it is unclear how endostatin protein itself affects microbial differences. Overall, our data indicated that *Desulfovibrio*, *Parabacteroides*, and *Parasutterella* might be important candidates for explaining the relationship between “endostatin” and “gut microbiota.” Nevertheless, future studies are needed to correlate such intestinal microbiota more definitively with recombinant *B. longum-Endo*.

## Conclusion

In conclusion, we demonstrated that *B. longum-Endo* alleviated inflammation response by inhibiting the expression of pro-inflammatory factors in DSS-induced colonic inflammation. Moreover, long-term oral administration of *B. longum-Endo* efficiently inhibited CRC tumorigenesis and improved intestinal flora dysbiosis. Our study suggested that oral administration of recombinant *B. longum-Endo* strain may be a promising therapeutic strategy for IBD and colitis-associated cancer.

## Data Availability Statement

The data presented in the study are deposited in the NCBI Sequence Read Archive repository, accession number: PRJNA838451. Raw data is avaliable at this link: https://www.ncbi.nlm.nih.gov/sra/PRJNA838451.

## Ethics Statement

The animal study was reviewed and approved by Nanjing University Animal Care and Use Committee.

## Author Contributions

HZ, Z-CH, and G-XX designed the outline of the manuscript. HZ revised this manuscript. ZB performed most of the experiments in this study and wrote the manuscript. EC and YY helped with the recombinant strain construction-related experiments. XC, XW, and YZ helped with the experiments using animals. All authors have read and approved the final version of this manuscript.

## Conflict of Interest

Z-CH was director of Jiangsu Target Pharma Laboratories Inc. The remaining authors declare that the research was conducted in the absence of any commercial or financial relationships that could be construed as a potential conflict of interest.

## Publisher’s Note

All claims expressed in this article are solely those of the authors and do not necessarily represent those of their affiliated organizations, or those of the publisher, the editors and the reviewers. Any product that may be evaluated in this article, or claim that may be made by its manufacturer, is not guaranteed or endorsed by the publisher.

## Footnotes

1
http://img.jgi.doe.gov/

2
http://www.kegg.jp/

3
http://kiwi.cs.dal.ca/Software/STAMP
