# Supplementary material for: Recombinant Bifidobacterium longum Carrying Endostatin Protein Alleviates Dextran Sodium Sulfate-Induced Colitis and Colon Cancer in Rats
Source: Front Microbiol. 2022 Jun 30;13:927277. doi: 10.3389/fmicb.2022.927277 (PMC9280188; doi:10.3389/fmicb.2022.927277)
Supplement: Supplementary file 2 [file Data_Sheet_2.docx]

**Supplementary information**

**Recombinant *Bifidobacterium longum* Carrying Endostatin Protein Alleviates Dextran Sodium Sulfate-Induced Colitis and Colon Cancer in Rats**

Zhiqian Bi^1^, Enqing Cui ^1^, Yingying Yao^1^, Xiaoyao Chang^1^, Xiaoyang Wang^1^, Yuhui Zhang^1^, Gen-Xing Xu^1,*^, Hongqin Zhuang^1,*^, Zi-Chun Hua^1,2,3, *^

^1^The State Key Laboratory of Pharmaceutical Biotechnology, College of Life Sciences, Nanjing University, Nanjing, P. R. China

^2^Changzhou High-Tech Research Institute of Nanjing University and Jiangsu Target Pharma Laboratories Inc., Changzhou 213164, P. R. China

^3^School of Biopharmacy, China Pharmaceutical University, Nanjing, China

***Corresponding authors:**

Hongqin Zhuang, School of Life Sciences, Nanjing University, 163 Xianlin Blvd., Nanjing 210023, China. Phone: 86-25-89683692; Fax: 86-25-83324605, E-mail: [hqzhuang@nju.edu.cn](mailto:hqzhuang@nju.edu.cn)

Zi-Chun Hua, School of Life Sciences, Nanjing University, 163 Xianlin Blvd., Nanjing 210023, China. Phone: 86-25-89683692; Fax: 86-25-83324605, E-mail: hzc1117@nju.edu.cn

Gen-Xing Xu, School of Life Sciences, Nanjing University, 163 Xianlin Blvd., Nanjing 210023, China. Phone: 86-25-89683692; Fax: 86-25-83324605, E-mail: genxingx@yahoo.com.cn

**Supplementary Tables**

Supplementary Table S1. The sequences of the primers used for RT-PCR of mouse colorectal tissue samples

| Gene | Primer name | Sequence | length |
| --- | --- | --- | --- |
| β-actin | β-actin-F | 5′-GCACCACACCTTCTACAATGAG-3′ | 22 |
| β-actin | β-actin-R | 5′-TTGGCATAGAGGTCTTTACGGA-3′ | 22 |
| ZO-1 | ZO-1-F | 5′-CTTCTCTTGCTGGCCCTAAAC-3′ | 21 |
| ZO-1 | ZO-1-R | 5′-TGGCTTCACTTGAGGTTTCTG-3′ | 21 |
| Occludin | Occludin-F | 5′-CACACTTGCTTGGGACAGAG-3′ | 20 |
| Occludin | Occludin-R | 5′-TAGCCATAGCCTCCATAGCC-3′ | 20 |
| IL-1β | IL-1β-F | 5′-TGGACCTTCCAGGATGAGGACA-3′ | 22 |
| IL-1β | IL-1β-R | 5′-GTTCATCTCGGAGCCTGTAGTG-3′ | 22 |
| TNF-α | TNF-α- F | 5′-GGTGCCTATGTCTCAGCCTCTT-3′ | 22 |
| TNF-α | TNF-α- R | 5′-GCCATAGAACTGATGAGAGGGAG-3′ | 23 |
| IL-6 | IL-6-F | 5′-TACCACTTCACAAGTCGGAGGC -3′ | 22 |
| IL-6 | IL-6-R | 5′- CTGCAAGTGCATCATCGTTGTTC-3′ | 23 |

Supplementary Table S2. Organ parameter changes in mice at 7 days

| Group | Body weight | Organ indices (weight/ body weight x 100) | | | Length (cm) | |
| --- | --- | --- | --- | --- | --- | --- |
|  |  | Liver | Spleen | Kidney | Small intestine | Colon |
| Control | 25.86$\pm$0.64 | 5.25$\pm$0.15 | 0.26$\pm$0.0084 | 1.13$\pm$0.04 | 39.3$\pm$1.90 | 8.73$\pm$0.22 |
| Model | 19.99$\pm$0.39** | 5.17$\pm$0.08 | 0.29$\pm$0.01* | 1.1$\pm$0.04 | 33.4 ±1.98* | 5.16$\pm$0.13** |
| B.longum | 22.83$\pm$0.16**,†† | 5.27$\pm$0.01 | 0.27$\pm c$0.01† | 1.17$\pm$0.03† | 37.38$\pm$1.25*,† | 7.01$\pm$0.19† |
| B.longum-Endo | 22.09$\pm$0.45**,† | 5.24$\pm$0.11 | 0.27$\pm$0.01† | 1.17$\pm$0.04 | 37.12$\pm$1.51*,† | 6.65$\pm$0.18**,†† |

Values are expressed as the mean ± SD, n=6-8;

* significantly different from the corresponding control group at 0.05 level;

** significantly different from the corresponding control group at 0.01 level;

† significantly different from the corresponding model group at 0.05 level;

†† significantly different from the corresponding model group at 0.01level.

**Supplementary Figures**

**
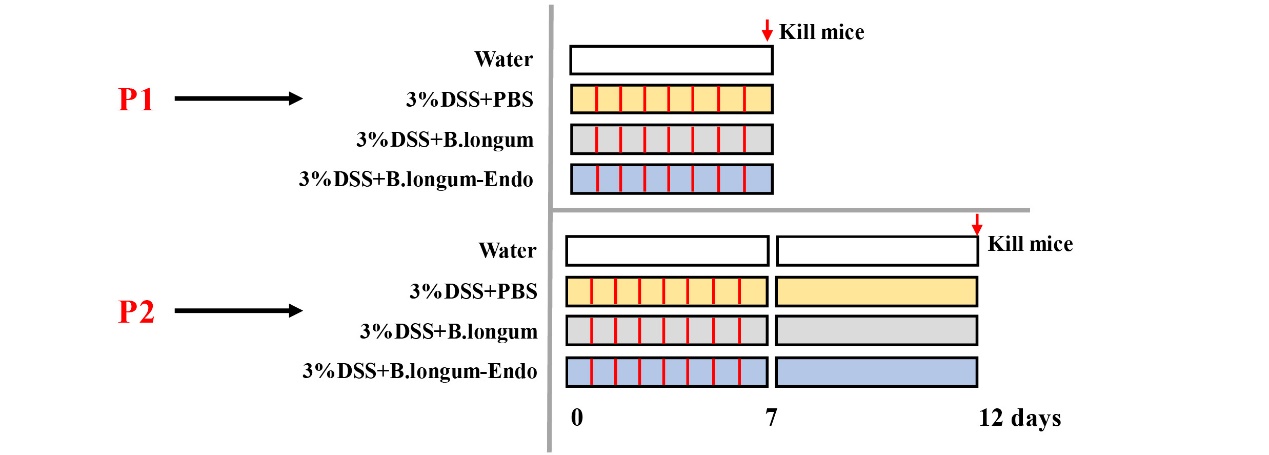
**

**Supplementary Figure S1.** Schematic representation of the DSS-induced mouse model of colitis. P1: Mice were treated with 3% DSS in drinking water for 7 days to induce acute colitis. During this week, *B.longum* or *B.longum-Endo* administration was continued by oral gavage. P2 represents a recovery experiment protocol: Mice were treated with 3% DSS in drinking water for 7 days and then changed to sterile water for additional 5 days. Oral administration with *B.longum* or *B.longum-Endo* was performed once daily for 12 days.


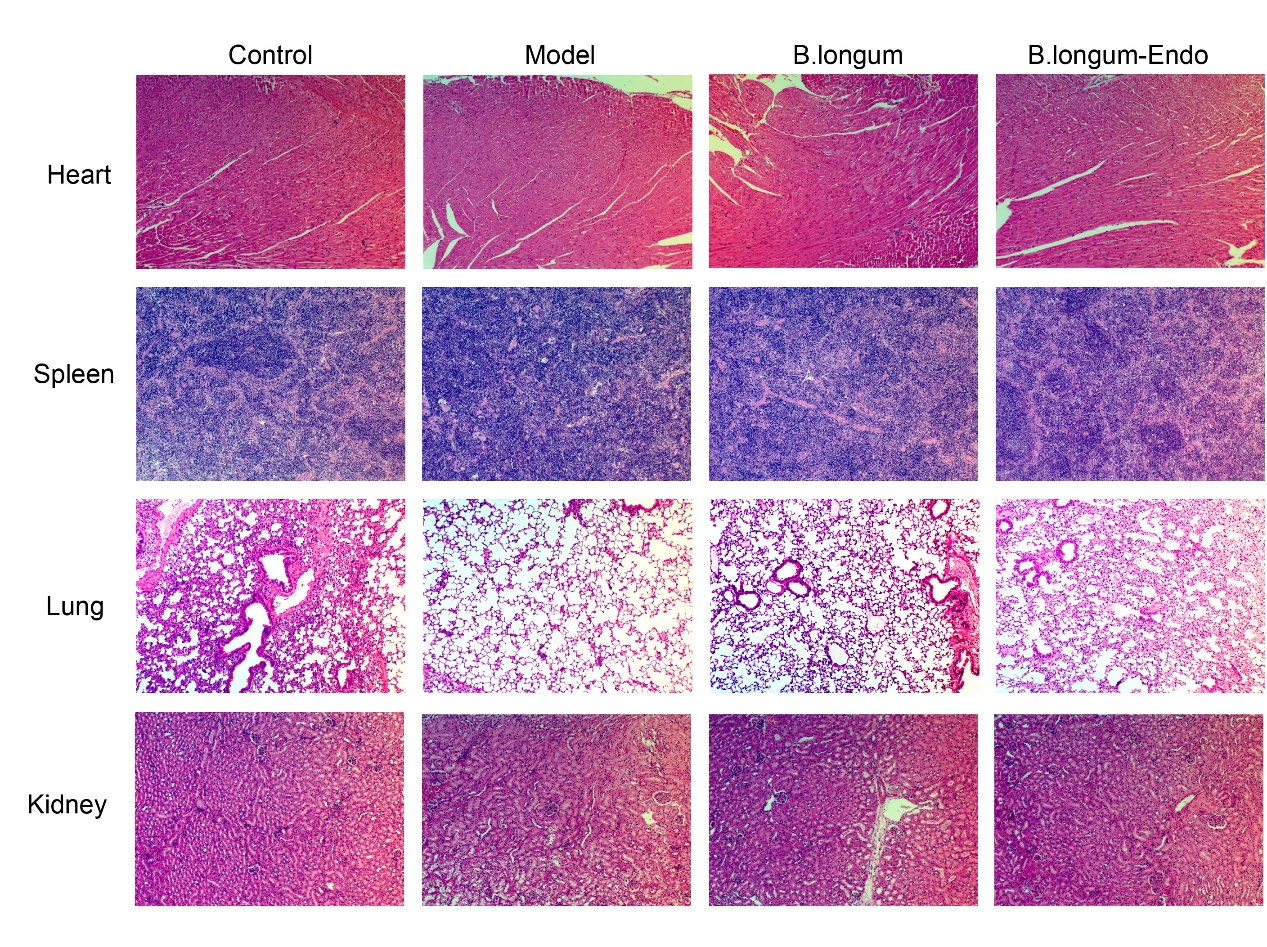
**Supplementary Figure S2.** Representative hematoxylin & eosin-stained sections of the major organs (heart, spleen, lung, kidney).

**
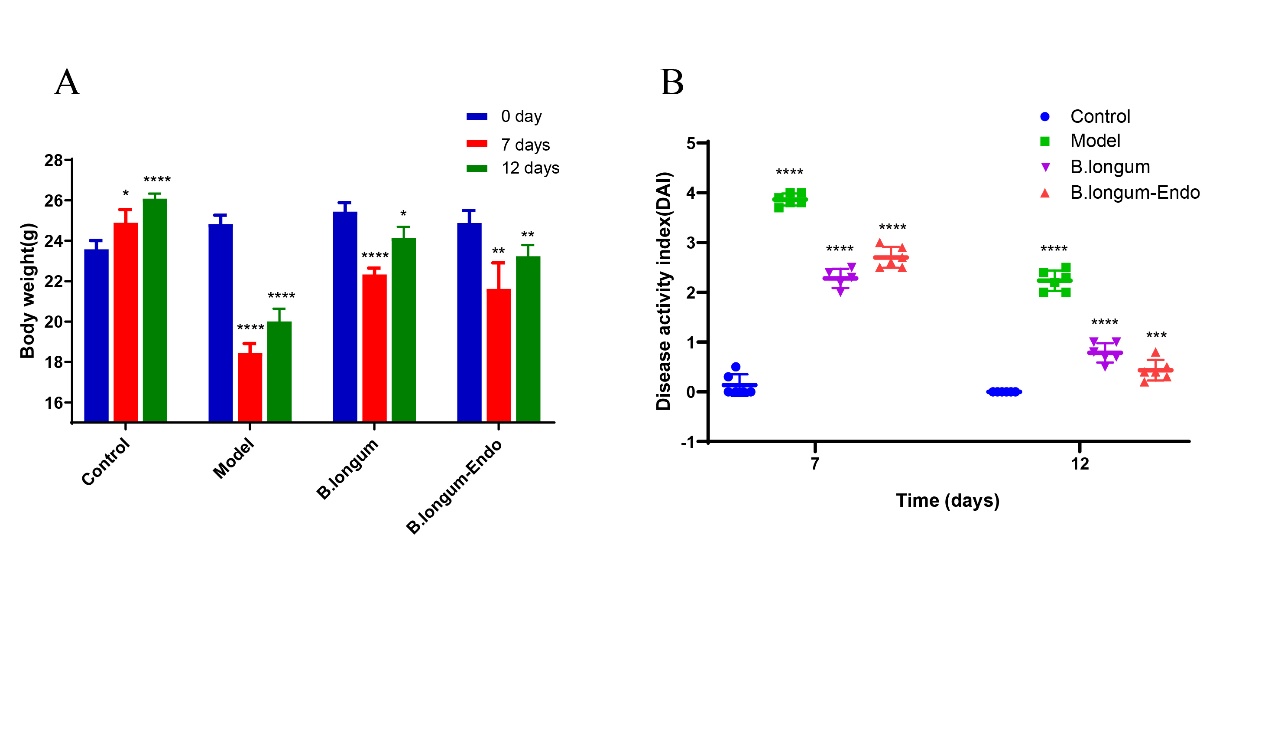
**

**Supplementary Figure S3. (A)** Body weight change (days 0, 7, 12); **(B)** Disease activity index score, a composite measure of weight loss, stool consistency and blood in stool (days 7, 12). Data are expressed as mean ± SD of three independent experiments (n=6). **P* < 0.05, ***P* < 0.01, ****P* < 0.001, *****P* < 0.0001, ns: not significant.


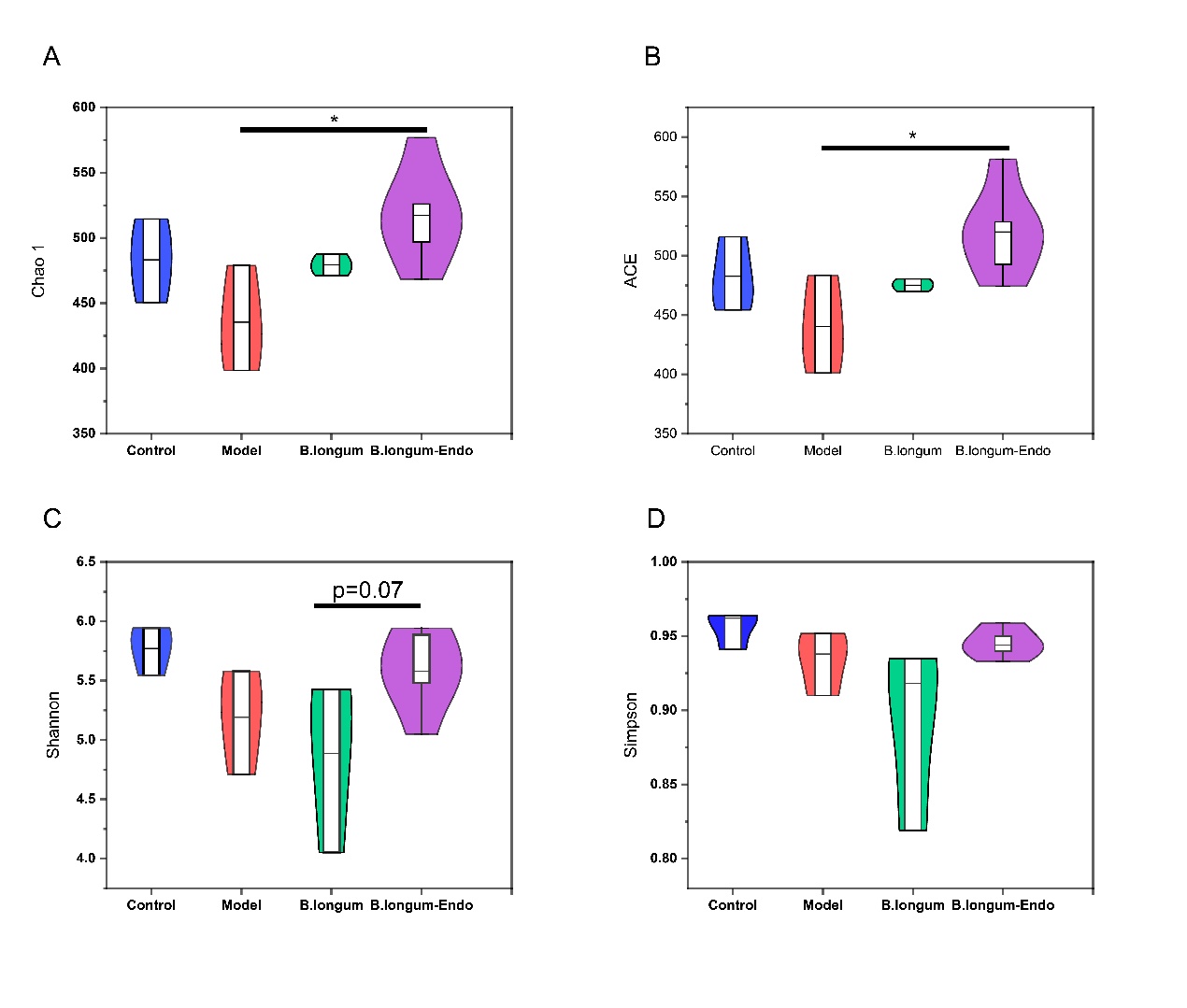


**Supplementary Figure S4.** Alpha diversity analysis of the differential microbial community among the Control, Model group, *B. longum group*, *B. longum-Endo* group. (A) Chao 1 index; (B) ACE index; (C) Shannon index; (D) Simpson index. **P* < 0.05.

**
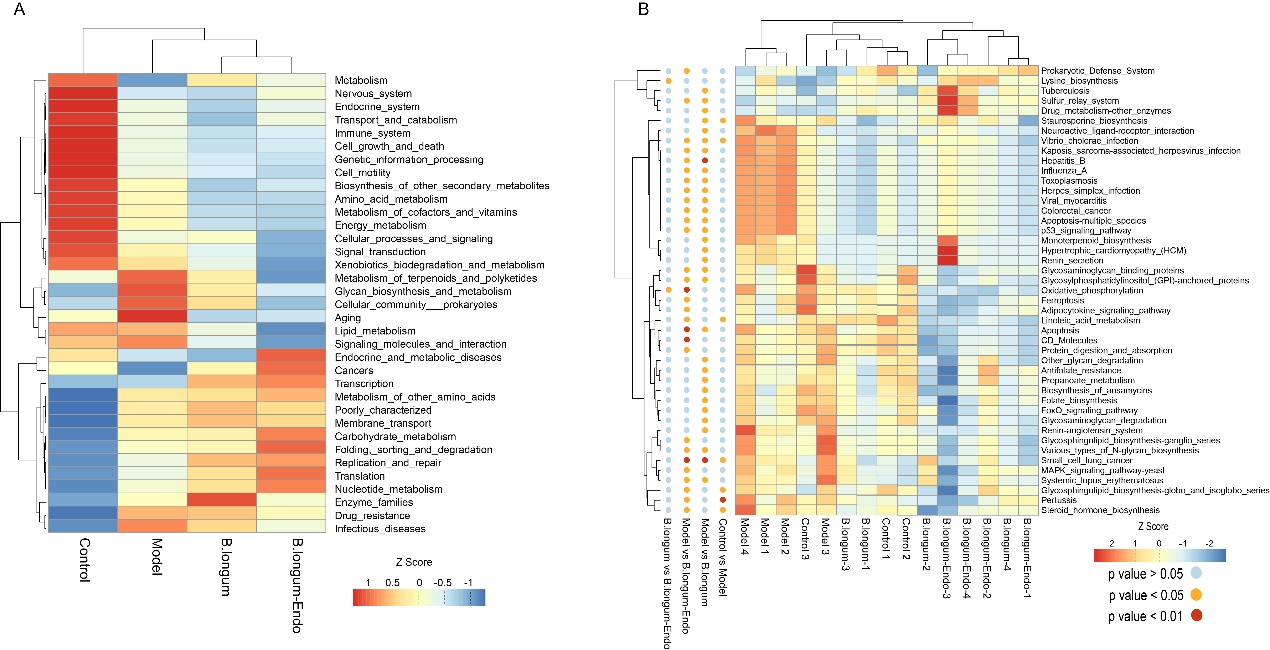
**

**Supplementary Figure S5.** KEGG orthologous gene cluster (KO) abundance heat map combined with cluster analysis. Heatmap showed relative bacterial abundances and associated function orthologs in level two (A) and three (B) KEGG pathways.
